# Real-Time Corrosion Monitoring of AISI 1010 Carbon Steel with Metal Surface Mapping in Sulfolane

**DOI:** 10.3390/ma12193276

**Published:** 2019-10-08

**Authors:** Andrzej Bak, Bozena Losiewicz, Violetta Kozik, Julian Kubisztal, Paulina Dybal, Aleksandra Swietlicka, Krzysztof Barbusinski, Slawomir Kus, Natalia Howaniec, Josef Jampilek

**Affiliations:** 1 Institute of Chemistry, University of Silesia, Szkolna 9, 40-007 Katowice, Poland; violetta.kozik@us.edu.pl (V.K.); pdybal@us.edu.pl (P.D.); aswietlicka@us.edu.pl (A.S.); 2 Institute of Materials Science, University of Silesia, 75 Pulku Piechoty 1A, 41-500 Chorzow, Poland; bozena.losiewicz@us.edu.pl (B.L.); julian.kubisztal@us.edu.pl (J.K.); 3 Institute of Water and Wastewater Engineering, Silesian University of Technology, Konarskiego 18, 44-100 Gliwice, Poland; krzysztof.barbusinski@polsl.pl; 4 Honeywell Process Solutions, 11201 Greens Crossing Blvd, Suite 700 Houston, TX 77067, USA; 5 Department of Energy Saving and Air Protection, Central Mining Institute, Plac Gwarkow 1, 40-166 Katowice, Poland; nhowaniec@gig.eu; 6 Department of Analytical Chemistry, Faculty of Natural Sciences, Comenius University, Ilkovicova 6, 842 15 Bratislava, Slovakia

**Keywords:** aprotic solvent, carbon steel, low-conductivity corrosion rate, electrochemical techniques, real-time corrosion monitoring, sulfolane

## Abstract

Solvents are widely used in organic synthesis. Sulfolane is a five-membered heterocyclic organosulfur sulfone (R-SO_2_-R’, where R/R’ is alkyl, alkenyl, or aryl) and an anthropogenic medium commonly used as industrial extractive solvent in the liquid-liquid and liquid-vapor extraction processes. Under standard conditions sulfolane is not aggressive towards steel, but at higher temperatures and in oxygen, water, or chlorides presence, it can be decomposed into some corrosive (by-)products with generation of SO_2_ and subsequent formation of corrosive H_2_SO_3_. This pilot-case study provides data from laboratory measurements performed in low conductivity sulfolane-based fluids using an industrial multi-electrochemical technique for reliable detection of corrosion processes. In particular, a comprehensive evaluation of the aqueous phase impact on general and localized corrosion of AISI 1010 carbon steel in sulfolane is presented. Assessment of corrosive damage was carried out using an open circuit potential method, potentiodynamic polarization curves, SEM/EDS and scanning Kelvin probe technique. It was found that an increase in the water content (1–3 vol.%) in sulfolane causes a decrease in the corrosion resistance of AISI 1010 carbon steel on both uniform and pitting corrosion due to higher conductance of the sulfolane-based fluids.

## 1. Introduction

A significant part of indoor or outdoor pollution is caused by volatile organic compounds (VOCs) and inorganic odorous compounds (VIOCs) that pose hazards to human health and plant vegetation [1]. The odor-producing mixtures of sulfur-based pollutants form a relevant group of contaminants with potentially detrimental impact on human health as a result of long-term exposure [2]. In fact, a wide range of environmental contaminants was designed rationally and engineered specifically for industrial targets. The process of eliminating the acidic gases (composed of H_2_S/CO_2_ mixture) to ‘soften’ the natural/industrial (off)gases is called gas treatment [3]. Growing public awareness of the need for environmental protection is the major driving force behind more rigorous regulations concerning release of hazardous pollutants and reduced sulfur compounds (RSCs) as well [4]. As a consequence, the removal of sulfur-containing compounds from unprocessed natural gases can be achieved by a liquid-liquid extraction process with closed loop application of industrial solvents (on-site recovery and regeneration) [5]. In this context, the questions about ‘green’ solvents and manufacturing procedures for the liquid-liquid extraction are quite important. An attractive alternative to heavily used extractive liquids is sulfolane (C_4_H_8_SO_2_), an anthropogenic organosulfur medium commonly used in industry due to its physicochemical properties [6,7].

### Beware of Sulfolane

Due to its satisfactory selectivity, low boiling temperature, and its capacity for dissolving large quantities of aromatics, sulfolane is the preferred solvent for liquid-liquid and liquid-vapor extraction processes when separating compounds with various degrees of saturation and polarity in the extractive rectification of BTX (benzene, toluene, xylene) mixture from the non-aromatic saturated hydrocarbons [8,9,10].

**Figure 1:**
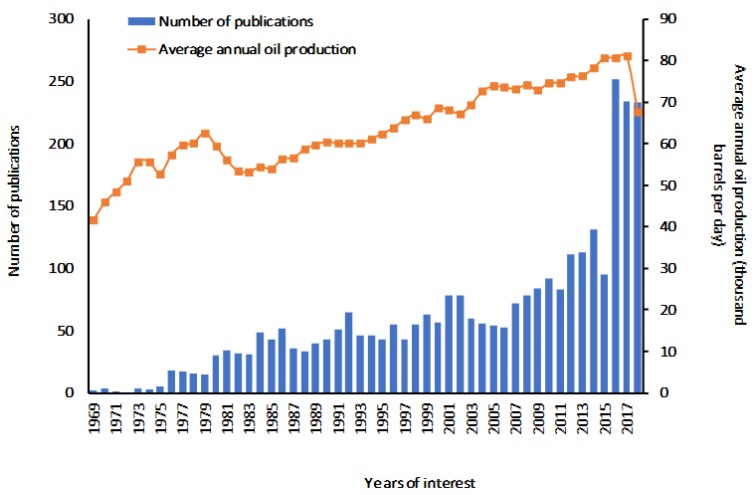
The average annual crude oil production and interest in sulfolane during the last five decades (from 1969 to 2018). Plot of the number of papers, where ‘sulfolane’ was searched in the paper title or abstract and the average annual oil production (thousand barrels/day) based on Reaxys and EIA data [11,12].

It is natural to ask the question what actually induced the growing interest in sulfolane that diminished after some years? Analysis of data stored in the commercially available Reaxys database [11] indicated a visible tendency of waxing and waning interest in sulfolane in the period of the last 50 years (from 1969 to 2018) (see Figure 1). Basically, 1644 hits were identified with the word ‘sulfolane’ in the title or abstract of the papers published in the last five decades—most of them describe sulfolane physicochemical properties (approx. 43%). Not surprisingly, the waves of interest correlate quite well with the general trends observed in the oil production market, as indicated in Figure 1.

Essentially, constant growth in the annual oil production observed throughout the last 50 years remains directly related to increased global oil consumption [12]. The indicated wavy trends in the sulfolane-related scientific activity partially correlate with annual oil production. It is noticeable that the plateau in the number of published papers can superimpose with the periods of relative stability in the annual oil production, as observed between 1984 and 1987 or towards the end of the first decade in the new millennium. It seems obvious that economic aspects of the aromatic extraction in the refining processes used in the oil industry do exert pressure on the scientific community.

Originally, the process of sulfolane synthesis involved three basic stages, culminating in hydrogenation of 3-sulfolene product of sulfur dioxide and butadiene reaction. Regarding the environmental constraints, introduction of the eco-friendly catalytic protocols promoted contemporary methods of sulfolane preparation assuring operational simplicity, rapid reaction rates, and effective formation of easily handled reagents [13]. Pollution-free catalysts recyclable under mild reaction conditions with halogen-free solvents and ‘green’ oxidants are an attractive alternative for large-scale industrial sulfolane production [14].

Theoretically, widespread industrial usage of sulfolane is disposal/leakage-free owing to its on-site recovery and regeneration; however, in practice some unpredicted/accidental spills, as well as leaks from extraction units in refineries or gas plants have been reported worldwide resulting in the soil and groundwater contamination [15]. In this context a question naturally appears concerning major reasons for accidental leaks and spills of sulfolane. Is it really important to monitor the sulfolane-induced corrosion of the industrial systems, knowing that pure sulfolane under standard operating conditions is considered to be a stable compound non-aggressive to steel? Unfortunately, the answer to this question is affirmative. Sulfolane-containing systems, when polluted by a small quantity of oxygen and under typical process requirements can be decomposed to form the corrosive (by-)products with generation of SO_2_ and subsequent formation of corrosive H_2_SO_3_ according to the following equation:

C_4_H_8_O_2_S + O_2_ + H_2_O → H_2_SO_3_ + C_3_H_7_CO_2_H. (1)

The corrosion of steel can be quite rapid, causing severe damage to industrial installations. Therefore, sulfolane should be stored under a nitrogen blanket and out of contact with oxygen, water, and strong oxidizing agents such as chlorates, nitrates, and peroxides, otherwise the solvent can be decomposed. Moreover, the presence of impurities such as oxygen and/or chlorides or water can accelerate sulfolane breakdown process as well. Pure (100%) sulfolane is not widely applied for aromatic extraction, especially in the Northern Hemisphere countries, due to its high viscosity at lower temperatures. Small quantities of water (1%–3%) usually added to sulfolane to assist plant transfer and storage operations may accelerate both general and localized corrosion due to higher conductance of the sulfolane fluid and hence easier ion transfer between electrochemical cells. Additionally, the presence of water may escalate the effect of halide impurities (mostly chlorides) on sulfolane corrosivity. Some general correlations between oxygen/chloride and water concentrations have been reported with sulfolane degradation and production of acidic corrodents leading to enhanced sulfolane corrosivity [16,17].

On-line, real-time corrosion monitoring of processes in low conductivity fluids like sulfolane aromatic extraction remain a challenge for traditional electrochemical techniques. Sulfolane specific conductance (typically about 5 µS/cm) excludes specifying the corrosion current by standard Linear Polarization Resistance (LPR) method, because the field of application has generally been limited by the need for a conductive/aqueous process environment. Hence, corrosion processes in sulfolane units are mostly monitored by time-lagging techniques like electrical resistance (ER). For corrosion measurements in low-conductivity fluids better performance has been achieved through application of multi-electrochemical approaches that synergistically combine low frequency impedance measurements (LFI), harmonic distortion analysis (HDA), and electrochemical noise (ECN), respectively [17]. In fact, it has been shown that real-time technique can be successfully employed for corrosion rate evaluation under various conditions [18] and compared with the post-test surface probe examination [19,20].

The real-time corrosion monitoring with the metal surface mapping procedures were combined together in the pilot workflow for the exhaustive assessment of the sulfolane corrosive potential against AISI 1010 carbon steel. The presented pilot-case study provides data from the laboratory measurements in low conductivity, sulfolane-based fluids utilizing the industrial, multi-electrochemical technique for reliable detection of the corrosion processes. A comprehensive evaluation is presented concerning impact which aqueous phase exerts on general and localized corrosion of AISI 1010 carbon steel in sulfolane. Several aspects of the corrosion measurement were taken into account, including the influence of process parameters (temperature as well as the impact of impurities, e.g., water, oxygen, and chlorides) on the corrosion of AISI 1010 carbon steel in pure sulfolane. Our incipient attempts to quantify the impact of process parameters (e.g., temperature) and impurities (e.g., water, oxygen, and chlorides) on AISI1 1010 carbon steel corrosion in pure sulfolane have provided some meaningful data [21,22]. The findings for AISI 1010 carbon steel demonstrate applicability of multi-technique electrochemical monitoring systems for rapid and accurate detection of corrosion in sulfolane. In this work, an attempt to assess corrosive damage of AISI 1010 carbon steel after real-time corrosion monitoring at 95 °C in sulfolane without and with the addition of 1–3 vol.% water was also made using open circuit potential (*E*_OC_) measurements, potentiodynamic polarization curves, scanning electron microscopy along with energy dispersion spectroscopy (EDS) and scanning Kelvin probe (SKP) technique. A micro-analytical technique of EDS conventionally used in SEM was also applied for local determination of chemical element distribution in the tested samples. It is the first potentiodynamic study on AISI 1010 carbon steel in low-conducting sulfolane-based solutions, determination of corrosion resistance parameters on this basis using the Tafel extrapolation method and the method of corrosion damage assessment based on the SEM images subjected to a numerical analysis, determination of contact potential difference and surface maps of the investigated materials by means of Scanning Kelvin Probe technique as well. The application of the ohmic drop compensation for the potentials recorded in the low-conductivity sulfolane and fitting the Butler–Volmer equation to Tafel plots using a numerical method was necessary to perform the analysis of polarization curves. The fitted polarization curves were the basis for the specification of the corrosion potential, the corrosion current density, the anodic and cathodic Tafel slopes.

The results of this pilot case study strongly encourage more extensive investigation including a wider range of solution conditions and the corresponding electrode surface analysis, respectively.

## 2. Materials and Methods

### 2.1. Design of Laboratory-Scale System

**Figure 2:**
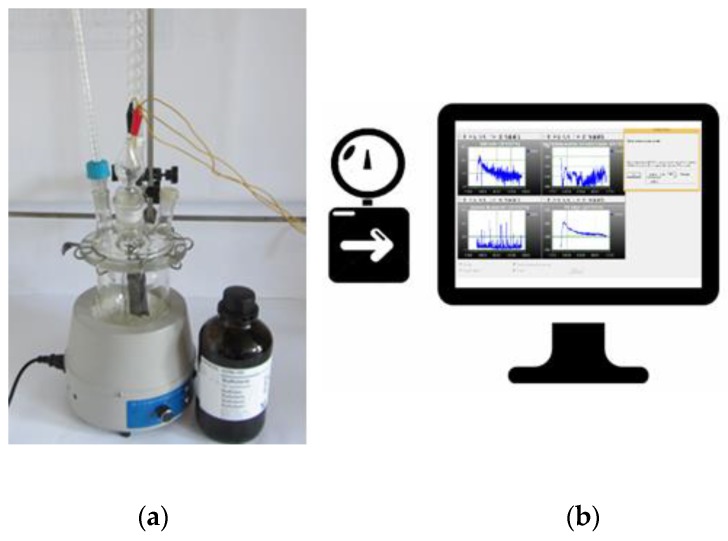
The laboratory-scale setup for (**a**) sulfolane corrosivity evaluation with (**b**) the corrosion data logger system.

The main purpose of the described pilot-scale study conducted on AISI 1010 carbon steel was to analyze selected factors that could potentially affect the low-conductivity corrosion rate. Monitoring of specific parameters allows to determine trends, similarities, and differences between general and local corrosion mechanisms. Laboratory tests regarding the impact of process parameters (temperature) and pollutants, for instance water content, on the corrosion of carbon steel in pure sulfolane provided important data and clues that may find practical application in the large-scale industrial processes. A dedicated test vessel was designed and constructed for performing the proposed investigations. Electrodes were supported on a special grooved glass frame to ensure their stability during the experiment and maintenance of contact with the solvent. Electrodes were separated by additional glass spacers. The electrodes were immersed in a round bottom, thick-walled reactor vessel of 500 cm^3^ capacity, made of heat-resistant glass. The test vessel was also equipped with mercury thermometer and a reflux condenser with a moisture absorber filled with CaCl_2_. The overall corrosion-test setup is shown in Figure 2.

To limit the contact of liquid with air, special silicone-type grease was used on all glass connections. For additional protection against the air ingress, each experiment was conducted under inert gas (99.995% Argon) blanket. The test vessel was placed in a heating bowl and heated to the appropriate temperature. During experiment sulfolane solution was continuously stirred (c.a. 1000 rpm, 1 cm magnetic stirrer) to simulate flowing conditions. We applied the SmartCET^®^ by Honeywell whose operation is based on Low Frequency Impedance (LFI), Harmonic Distortion Analysis (HDA) and Electrochemical Noise (ECN). The industrial, wired transmitter (model CET5500) was used in conjunction with the HART (Highway Addressable Remote Transducer) system. The set of three, flat coupon electrodes made of AISI 1010 carbon steel was also used (dimensions: 89 mm × 20 mm × 2 mm) in the study. Several aspects of the measurement of general and localized corrosion modes were scrutinized extensively.

### 2.2. Sulfolane Corrosivity Parameters

Each parameter, e.g., general corrosion rate, local corrosion potential (Pitting Factor), current Stern–Geary coefficient—B value—and Corrosion Mechanism Index (CMI) were measured and recorded at 60-s intervals using an appropriate data recording system. A brief description of the measured parameters was provided by NACE International (see Table 1) [23].

The experiment time length was set to 96 h on the non-standard electrodes made of AISI 1010 carbon steel of *ρ* = 7.86 g cm^−3^ density. A series of corrosion tests were carried out maintaining the process temperature in the range of 95 °C, 180–190 °C, and 230–240 °C with the pure sulfolane [17,21]. During the experiments with the addition of oxygen, air was introduced for 3 days under the surface of the sulfolane solution in appropriate quantities. The influence of chlorides (50 ppm) on the steel corrosion was examined as well. After each experiment, the system was cooled and disassembled and the electrodes were put into the dryer for 1 h. After 10 min of cooling, electrodes were weighed and then etched to remove corrosive products. The etching mixture was made with 100 cm^3^ of fuming HCl acid (38%), 5 g of SnCl_2_, and 2 g of Sb_2_O_3_ mixed in an ultrasound bath for 15 min. The electrodes were immersed in 50 cm^3^ of the mixture for 5 min. After etching, the electrodes were placed in the dryer for half an hour and then weighed three times after cooling. The whole procedure was repeated three times. The etching and calculation of the mass loss was carried out after each change in the parameters of the experiment.

**Scheme 1:**
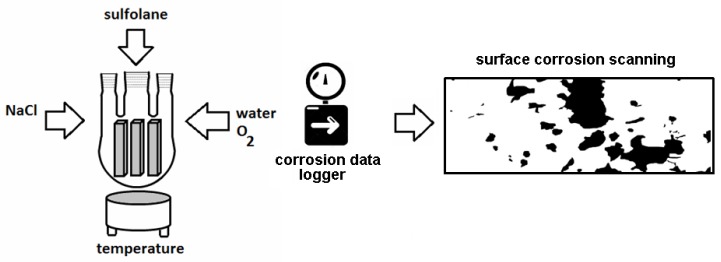
Schematic design of experiments.

A detailed description of each of the conducted experiments (see Scheme 1) is beyond the scope of this paper; therefore, we focused on the introduction of the selected water case study. It is expected that increasing water concentration in sulfolane will enable better ionic transfer between surface corrosion cells and hence may accelerate corrosion rate.

### 2.3. Surface Corrosion Evaluation

Assessment of corrosive damage of AISI 1010 carbon steel was carried out on the samples S1–S5 (see Table 2). 

Electrochemical corrosion studies were conducted in a three-electrode system in sulfolane at 25 °C using the *E*_OC_ method and potentiodynamic polarization curves. The sample under study was the working electrode with a geometric surface area of 8 cm^2^. Counter electrode was a Pt foil. All values of the potential were measured relative to the reference electrode in the form of saturated calomel electrode (SCE) connected to the sulfolane using Luggin capillary set at a distance of 2 mm from the cathode surface. Electrochemical studies were carried out using a computer-controlled electrochemical system ECO CHEMIE Autolab/PGSTAT12. The *E*_OC_ was stabilized for 120 min. Then the polarization curve of log |*j*| = f(*E*) in a narrow range of potentials corresponding to the cathode/anodic transition was recorded at the polarization rate of the electrode *v* = 1 mV s^−1^. The obtained curve was the basis for determination of the parameters of corrosion resistance of the tested samples such as corrosion potential (*E*_cor_), corrosion current density (*j*_cor_), anodic (*b*_a_), and cathodic (*b*_c_) Tafel slopes, respectively. Stern–Geary constant (*B*), polarization resistance (*R*_p_), corrosion rate (*CR*) at the *E*_cor_ and ohmic drop, were calculated according to ASTM G102-89(2015)e1: Standard Practice for Calculation of Corrosion Rates and Related Information from Electrochemical Measurements. The *B* constant was calculated according to the following equation:

(2) $$B=\frac{b_{a}\cdot b_{c}}{2.303(b_{a}+b_{c})}$$

The *R*_p_ was calculated from the corrosion current density and the *B* constant as follows:

(3) $$j_{\mathrm{cor}}=\frac{B}{R_{p}}$$

The corrosion rate calculations in terms of penetration rate were carried out based on the following equation:

(4) $$CR=K_{1}\frac{j_{\mathrm{cor}}}{\rho }EW,$$

where *CR* is given in mpy, *j*_cor_ in μA cm^−2^, *K*_1_ = 0.1288 in mpy g μA^−1^ cm^−1^, *ρ* in g cm^−3^, and the equivalent weight (*EW*) for iron (valence 2) equal to 27.923 was considered dimensionless. In these calculations it was assumed that the process of oxidation is uniform and does not occur selectively to any component of the alloy.

The measurement of conductivity of the sulfolane at 25 °C was performed using the CC-505 Laboratory Conductometer (ELMETRON). Anodic polarization curves were registered starting at 150 mV negative to the *E*_OC_ (*v* = 1 mV s^−1^) and proceeding in a forward direction through the cathode/anodic transition and continuing on to 4 V.

Surface morphology observations of electrodes following potentiodynamic measurements were carried out by means of a JEOL JSM-6480 Scanning Electron Microscope (SEM, Tokyo, Japan), equipped with an energy dispersive spectroscopy (EDS) system to measure chemical composition locally. The SEM images were converted to a black and white representation with black areas demonstrating surface of the corrosion pits. Then, using a numerical method, the total surface of pits, *S*_b_, and the total area of the image, *S*_t_ were determined. The corrosion degree (*CD*) was calculated according to the following equation:

(5) $$CD=\frac{S_{b}}{S_{t}}\cdot 100\%.$$

Contact potential difference (*CPD*) maps and surface maps of the investigated materials were recorded by means of Scanning Kelvin Probe (SKP) technique using PAR Model 370 Scanning Electrochemical Workstation (Odessa, FL, USA) equipped with a tungsten Kelvin probe (KP) with a diameter of 500 μm [24,25,26,27,28]. The scanning area was 4000 × 4000 μm^2^ and the distance between the probe and the sample was ca. 100 μm [29,30]. Histograms of the *CPD* distribution were created by dividing the range of *CPD* into 25 equal intervals (Δ*CPD*) and by determining the number of *CPD* (*n_i_*) lying in the range of each interval. In that way, the probability density function could be approximated by $n_{i}/\underset{i}{\sum }n_{i}\Delta CPD$.

## 3. Results and Discussion

To visualize and interpret the results, two software scripts were implemented in the MATLAB environment for downloading and analyzing data from a text file. Data pre-processing (binning) was used to show more accurately the trends of measured values; the average value was calculated from each 1-h interval. The implemented software scripts and operational configuration of the laboratory-scale equipment were applied to monitor the water content (1, 2, and 3 vol.%) and corrosion parameters such as general corrosion rate (PV—primary variable) expressed in mils per year (mpy), localized corrosion potential (dimensionless Pitting Factor), actual Stern–Geary coefficient—B value (mV)—and Corrosion Mechanism Indicator (CMI A cm^−2^). It should be emphasized that pure sulfolane used for experiments contained less than 0.2 vol.% of water; however, it was of interest to analyze the impact of water content on AISI 1010 carbon steel corrosion processes. As under extreme experimental conditions (temperature >230 °C) water would evaporate, we decided to conduct our experiments at temperature approx. 95 °C. Initial attempts to quantify the impact of impurities (water content) on AISI 1010 carbon steel corrosion in sulfolane solvent and relevant results are detailed below.

### 3.1. Pilot Study of Sulfolane-Induced Corrosion

**Figure 3:**
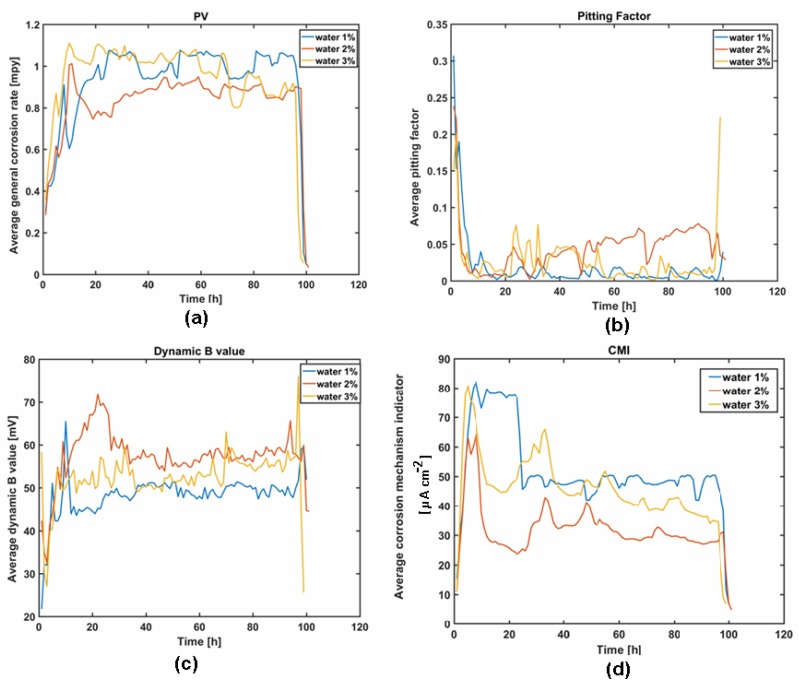
General corrosion rate (**a**), pitting factor (**b**), dynamic B value (**c**), and corrosion mechanism indicator (**d**) measured in sulfolane solutions with the addition of 1, 2, and 3 vol.% of water at 95 °C.

Surprisingly, the general corrosion rate did not increase with the amount of water in sulfolane solution; however, some trends were easily observed. It is evident that in the first 15 h of each experiment the average PV parameter was increased and subsequently stabilized with approx. 0.2 mpy fluctuations, as illustrated in Figure 3a.

We confirmed some earlier general correlations between water concentration and sulfolane corrosivity, where water above concentration of 3% was reported to accelerate sulfolane degradation and production of acidic corrodents [16,17]. It should be emphasized that the highest corrosivity recorded at 3% water concentration was still at an acceptable level. Moreover, the observed water-sulfolane trends were relatively stable and showed approximately the same variations over the experiment duration. General corrosion remained the dominant type of damage, irrespective of water concentration that was manifested by low levels of the Pitting Factor parameter, as depicted in Figure 3b. For all experiments with water-containing sulfolane the localized corrosion potential, expressed by Pitting Factor, was at very low level (PF << 0.1). It confirmed uniform corrosion of electrodes without macroscopic indications of pits. In other words, water concentration (1%–3% volume range) did not an exert impact on localized corrosion. After initial peaks all trends of the PF parameter were stabilized below 0.1 level indicating domination of the general corrosion mechanism for all considered sulfolane-water configurations. The B value (see Figure 3c) was quite stable during the experiments irrespective of water content; however, the presence of water (ca. 1 vol.%, at 95 °C), generated the lowest level of corrosion. Stability of high dynamic B value during long-term experiment can be related to the passive state of AISI 1010 carbon steel in water-containing sulfolane solutions. The CMI parameter (related to surface capacitance) was noticeably below 80 µA cm^−2^, which indicated absence of any significant deposition processes. After the initial fluctuations the CMI parameter for the experiments with water addition did not exceed 50 µA cm^−2^ (see Figure 3d), which means that no surface layer was formed during the measurement. On the other hand, under conditions with extremely low corrosion current values, CMI may not be used as a reliable indicator of surface-occurring processes.

An interesting observation was made regarding sulfolane color during the experiments in the sulfolane-water system. The more water in the solution, the more black sediment was observed. It seems a bit surprising since all experiments were performed at about 95 °C, that is far below sulfolane decomposition temperature (200–230 °C). Apparently, presence of water accelerates decomposition reactions, even at relatively low temperatures, causing more suspended solids but also increasing the corrosion level.

### 3.2. Electrochemical Methods for Corrosion Assessment

**Figure 4:**
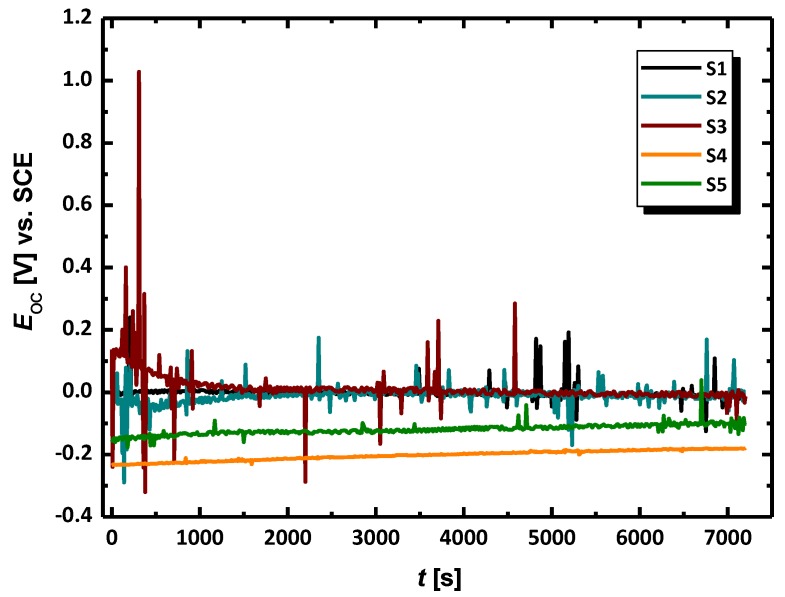
Open circuit potential for the AISI 1010 carbon steel electrode in sulfolane at 25 °C for S1 electrode after the experiment in pure sulfolane at 95 °C and etching; S2 electrode after the experiment in sulfolane with the addition of 1 vol.% of water at 95 °C and etching; S3 electrode after the experiment in sulfolane with the addition of 2 vol.% of water at 95 °C and etching; S4 electrode after the experiment in sulfolane with the addition of 3 vol.% of water at 95 °C and etching; and S5 electrode after the experiment in sulfolane at 95 °C and without etching of corrosion products.

The open circuit potentials for the AISI 1010 carbon steel electrodes studied in sulfolane at 25 °C for 120 min following the experiment at 95 °C are dependent on the surface treatment and water content in sulfolane solution (see Figure 4). It can be observed that the stable value of open circuit potential registered for S1 electrode, which was examined previously in pure sulfolane at 95 °C and etched in the mixture of HCl, SnCl_2_, and Sb_2_O_3_ in order to determine the mass loss by gravimetric method (*E*_OC_ = −7 mV), are close to the *E*_OC_ value obtained for S2 electrode after the experiment in sulfolane with the addition of 1 vol.% of water at 95 °C and etching (*E*_OC_ = 0 mV) and S3 electrode tested before in sulfolane with the addition of 2 vol.% of water at 95 °C and etched (*E*_OC_ = −13 mV). The ionic-electron equilibrium at the electrode|electrolyte interface for S4 electrode examined initially in sulfolane with the addition of 3 vol.% of water at 95 °C and subjected to etching, was attained at the most negative potential among all tested electrodes (*E*_OC_ = −178 mV). It should be noted that the etching procedure can influence the surface state of the investigated electrodes by passivation due to possibility of embedding of ingredients of the etching mixture into the surface. For the S5 electrode after the experiment in sulfolane at 95 °C and without etching, the *E*_OC_ was stabilized at a relatively low potential (−100 mV). It can suggest that in the latter case the corrosion products formed on the electrode surface during the experiment at 95 °C show poor protection properties. In Figure 4 one can observe strong fluctuations of potential for S2–4 electrodes examined in sulfolane containing water addition, which may probably be caused by breakdown of the passive layer and its repassivation, which occurs at the open circuit potential conditions and leads to the formation of metastable pits. The stabilized values of the *E*_OC_ from Figure 4 can be treated as approximate values of the *E*_cor_ for the AISI 1010 carbon steel electrodes in pure sulfolane at 25 °C.

**Figure 5:**
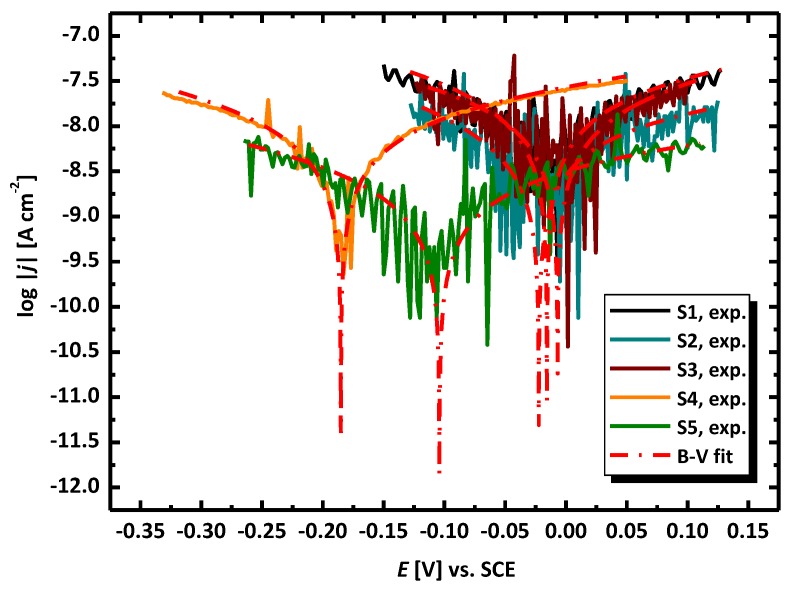
Tafel plots for the AISI 1010 carbon steel electrode in sulfolane at 25 °C showing experimental raw data (continuous line) and fitted data using the Butler–Volmer equation (dash dotted line) after prior compensation of the ohmic drop. S1-electrode after the experiment in pure sulfolane at 95 °C and etching, S2-electrode after the experiment in sulfolane with the addition of 1 vol.% of water at 95 °C and etching, S3-electrode after the experiment in sulfolane with the addition of 2 vol.% of water at 95 °C and etching, S4-electrode after the experiment in sulfolane with the addition of 3 vol.% of water at 95 °C and etching, and S5-electrode after the experiment in sulfolane at 95 °C and without etching of corrosion products.

The polarization curves registered in a narrow range of potentials relative to the stabilized *E*_OC_ value are shown in Figure 5. Experimental Tafel plots with raw data are presented using continuous lines. Dash dotted line denotes the data fitted using the Butler–Volmer (B-V) equation after prior compensation of the ohmic drop [31].

Determination of the corrosion resistance parameters for the AISI 1010 carbon steel electrodes in sulfolane at 25 °C based on the experimental raw data shown in Figure 5 was practically impossible due to the low conductivity of sulfolane, which was only 0.35 μS cm^−1^. Based on this parameter, the sulfolane resistivity was determined to be of 2.86 MΩ cm. The solution resistance (*R*_s_) was calculated as a product of the electrolyte resistivity and the distance between the specimen electrode and the Luggin capillary tip. The *R*_s_ was equal to 0.57 MΩ cm^2^. Such a high value of the *R*_s_ causes that even small currents can lead to large errors in the potential of the potentiodynamic measurements. Therefore, the impact of ohmic drop in experiments performed in low conductivity sulfolane is significant. For this reason, the ohmic drop compensation was applied, after which the shift of potentials on the curves log |*j*| = f(*E*) shown in Figure 5 towards more positive potentials was observed. Then, the Tafel plots with the compensated ohmic drop were fitted using a numerical method by means of the B-V equation used in the following form [28]:

(6) $$j=j_{\mathrm{cor}}\left\{\exp\left[\frac{2.303(E-E_{\mathrm{cor}})}{b_{a}}\right]-\exp\left[-\frac{2.303(E-E_{\mathrm{cor}})}{b_{c}}\right]\right\},$$

where *j* is electrode current density in A cm^−2^, *E* is electrode potential in V, *j*_cor_ is corrosion current density in A cm^−2^, *E*_cor_ is corrosion potential in V, *b*_a_ is anodic Tafel slope in V dec^−1^, and *b*_c_ denotes cathodic Tafel slope in V dec^−1^ [32]. The parameters of Equation (6) were fitted to the experimentally registered dependencies *j* = f(*E*) presented in Figure 5 and the values of *E*_cor_, *j*_cor_, *b*_a_, and *b*_c_ were determined and are presented in Table 3. It should be noted that the quality of the B-V fitting was very good. The *R*^2^ values were in the 0.990–0.999 range.

Corrosion potential is widely recognized as a parameter allowing initial assessment of corrosion properties of metals and alloys. This parameter allows to predict when the destructive processes will start in the tested material in a given corrosive environment. The determined *E*_cor_ values that correspond to the minima on polarization curves of log |*j*| = f(*E*) registered in the range of potentials close to the potential of the open circuit in Figure 5 indicate that electrode S4 has the lowest corrosion resistance in sulfolane at 25 °C (Table 3). This means that the addition of 3 vol.% of water to sulfolane at 95 °C caused the greatest acceleration of the corrosion process of AISI 1010 carbon steel due to increased electrolyte conductivity. Additionally, the lowest *E*_cor_ value equal to −0.185 V among all tested electrodes could be affected by the process of etching corrosion products and related changes in the surface morphology and the surface chemical composition of the S4 electrode.

The determined values of *j*_cor_ are directly proportional to the rate of electrochemical corrosion occurring, but they cannot be treated as a kinetic parameter for the comparative assessment of the corrosion resistance of the tested material (Table 3). All average values of corrosion current density for tested electrodes do not exceed 31 nA cm^−2^. At the same time, the smallest average *j*_cor_ value of approx. 4 nA cm^−2^ is observed for the S5 electrode, which before the potentiodynamic measurements in sulfolane at 25 °C was subjected to the experiment in pure sulfolane at 95 °C and the corrosion products were not removed from its surface. The obtained results may indicate that the present film of corrosion products on the surface of the S5 electrode is compact and exhibits protective properties, and the rate of its dissolution is slower than in the case of passive layers formed on the surface of AISI 1010 carbon steel during etching.

From the obtained Tafel slope values *b*_a_ and *b*_c_ one can draw conclusions about the mechanism of the electrode processes that are taking place (Table 3). The anodic process is the process of dissolving the alloy component. It can therefore be hypothesized that the occurring process is the dissolution of iron in accordance with the reaction:

(7) $$\mathrm{Fe}-2e^{-}\to {\mathrm{Fe}}^{2+}.$$

The cathodic process may be the reduction of hydrogen ions according to the equation:

(8) $$2H^{+}+2e^{-}\to H_{2}.$$

It should be noted that in all cases the electrodes tested, with the exception of the S3 electrode, *b*_a_ has higher values than *b*_c_, which means that the kinetics of the anode process is faster compared to the cathodic reaction rate. The reverse trend for the S3 electrode may result from the inhomogeneity of the electrode surface after etching.

From known Tafel slopes the Stern–Geary constants were calculated according to Equation (2) where both cathodic and anodic reactions were activation-controlled, which was confirmed by the presence of distinct linear regions near the corrosion potential on the Tafel plots in Figure 5. One can see that high *B* values in all studied cases indicate the passive state of the tested electrodes (Table 3).

The next determined parameter characterizing the AISI 1010 carbon steel resistance to electrochemical corrosion in sulfolane at 25 °C is the polarization resistance (Table 3). The average *R*_p_ values for electrodes subjected to prior tests at 95 °C in sulfolane without and with the addition of water (1–3 vol.%) and then etched, are high in the order of a few MΩ cm^2^. However, the highest polarization resistance of approx. 31 MΩ cm^2^ was determined in the case of the S5 electrode, from which surface the layer of corrosion products with strong barrier properties has not been removed. The calculated values of the *CR* at *E*_cor_ are presented in Table 3 and confirm the corrosion behavior of the tested electrodes, which was described using the calculated parameters *j*_cor_ and *R*_p_. The *CR* is an important quantitative parameter that allows to compare the corrosion properties of metals and alloys. Analyzing the calculated *CR* values for AISI 1010 carbon steel electrodes in sulfolane at 25 °C, it can be concluded that corrosion processes occur with the smallest intensity in the case of the S5 electrode, for which the penetration rate is 0.002 mpy.

**Figure 6:**
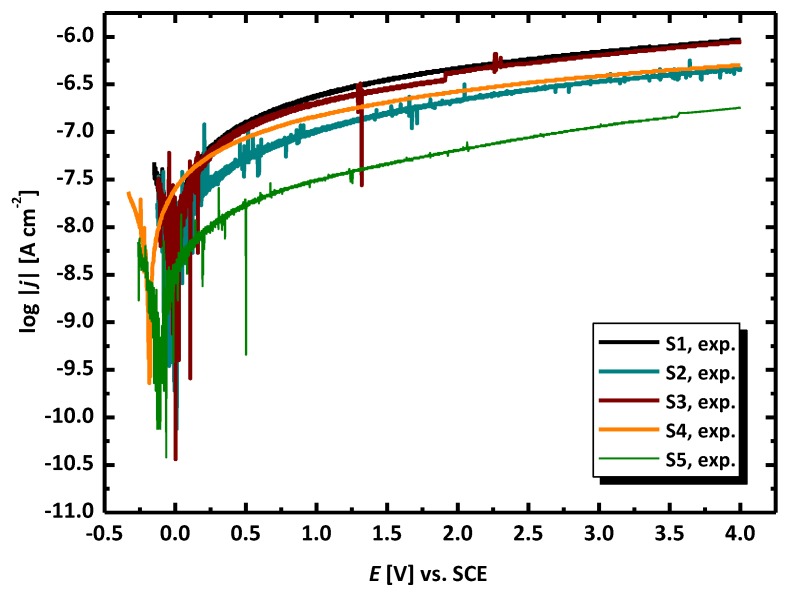
Anodic polarization curves for the AISI 1010 carbon steel electrode in sulfolane at 25 °C showing experimental raw data. S1-electrode after the experiment in pure sulfolane at 95 °C and etching, S2-electrode after the experiment in sulfolane with the addition of 1 vol.% of water at 95 °C and etching, S3-electrode after the experiment in sulfolane with the addition of 2 vol.% of water at 95 °C and etching, S4-electrode after the experiment in sulfolane with the addition of 3 vol.% of water at 95 °C and etching, and S5-electrode after the experiment in sulfolane at 95 °C and without etching of corrosion products.

Anodic polarization curves for the tested electrodes presented in Figure 6 reveal no typical plateau on the anodic branches corresponding to metal passivation/transpassivation. In the whole range of the anodic potentials studied the increase in anodic current density is visible with the increasing potential value; this is consistent with thermodynamic predictions, i.e., the higher the potential of the electrode the higher the current density recorded. However, it should be added that in the potential range of up to 4 V the tested materials are in the passive area and on the passive surface of the electrodes pits are formed. The anodic branch of the polarization curves show pit instability on the surface as current spikes.

### 3.3. Surface Corrosion Scanning

**Figure 7:**
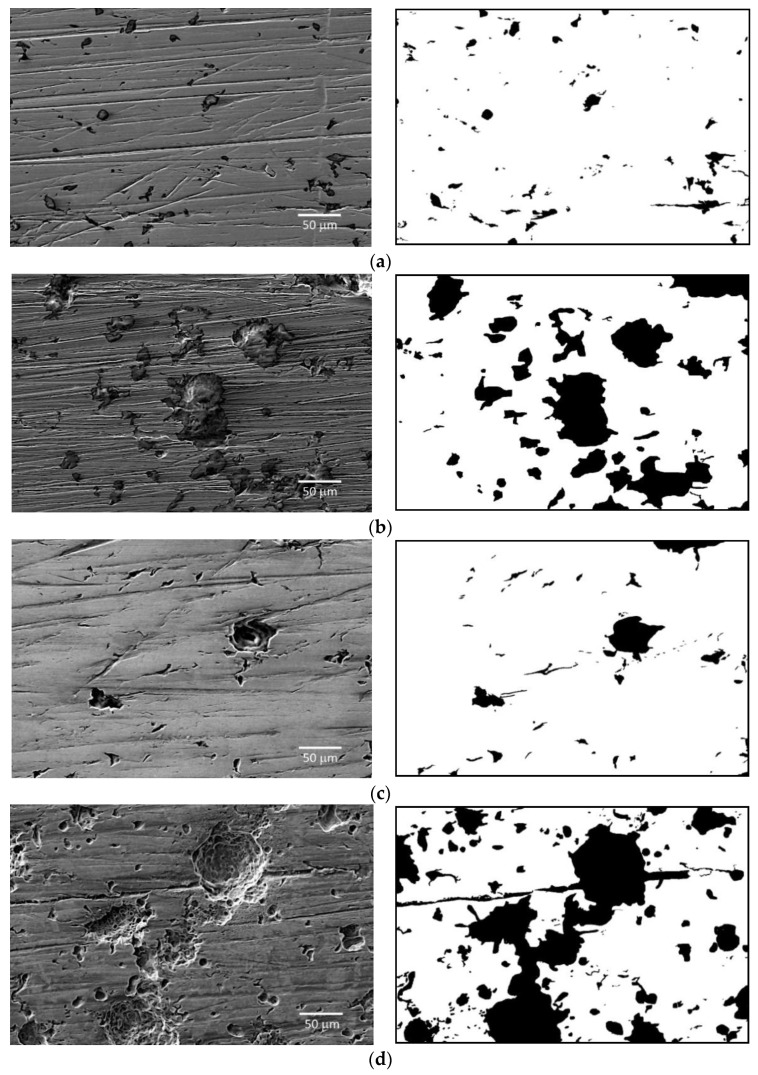
Exemplary SEM image (on the left) with the corresponding conversion to black and white (on the right) for the AISI 1010 carbon steel after the experiment in sulfolane at 95 °C without etching before (**a**) and after (**b**) potentiodynamic test; after the experiment in sulfolane at 95 °C with etching before (**c**) and after (**d**) potentiodynamic test (see Figure 6).

Assessment of the corrosion degree of AISI 1010 carbon steel electrodes subjected to the potentiodynamic measurements was performed using sample surface images obtained by SEM with the conversion to black and white, as illustrated in Figure 7.

It was determined that the degree of corrosion for the AISI 1010 carbon steel just after the experiment in sulfolane at 95 °C without etching was low (*CD* = 3%), as illustrated in Figure 7a. Only a slightly higher value (*CD* = 4%) was found for the sample after the experiment in sulfolane at 95 °C and etched, as shown in Figure 7c. However, in both cases the degree of corrosion strongly increased after the potentiodynamic tests up to *CD* = 20% (Figure 7b) and *CD* = 30% (Figure 7d), respectively.

**Figure 8:**
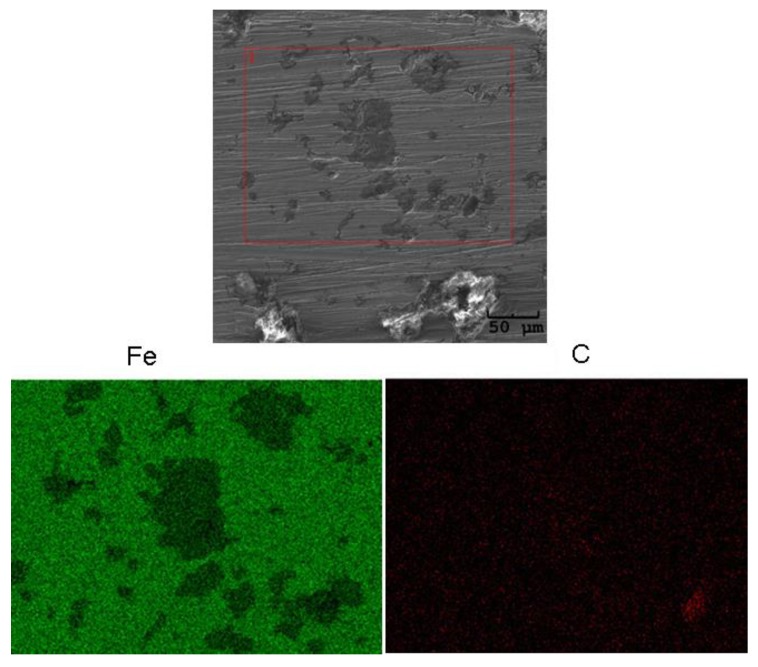
SEM image of the AISI 1010 carbon steel surface after the experiment in sulfolane at 95 °C without etching and the potentiodynamic test: on-top general view of the material with a selected micro-region and with the corresponding energy dispersion spectroscopy (EDS) map of Fe and C element distribution.

**Figure 9:**
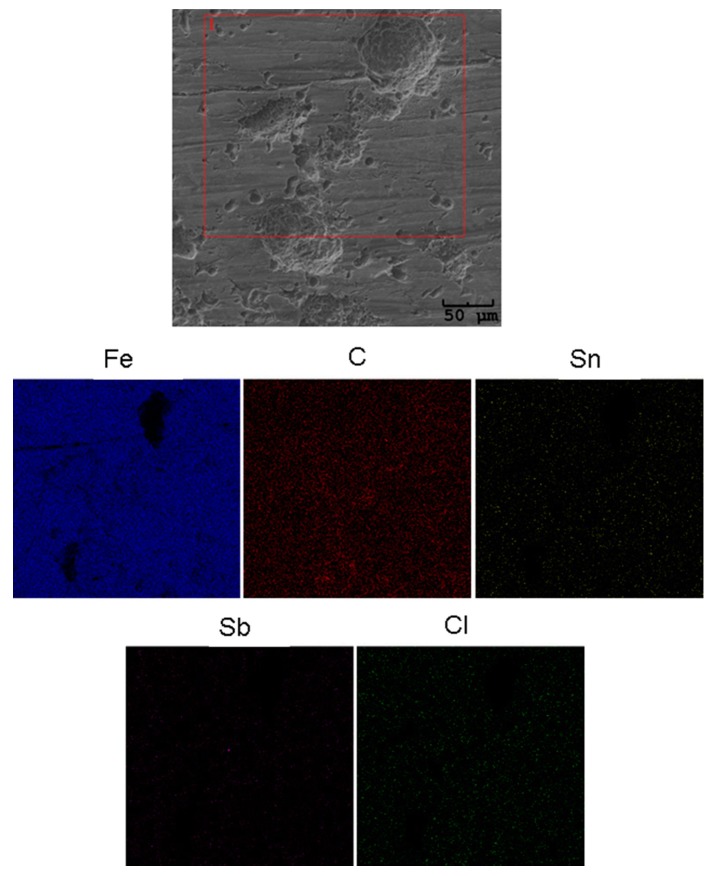
SEM image of the AISI 1010 carbon steel surface after the experiment in sulfolane at 95 °C and etchig, and then the potentiodynamic test: on-top general view of the material with a selected micro-region, and with the corresponding EDS map of Fe, C, Sn, Sb, and Cl element distribution.

The SEM observation of the microstructure of the AISI 1010 carbon steel without (see Figure 8 and with etching (see Figure 9) after the experiment at 95 °C and subjected to the potentiodynamic test in sulfolane at 95 °C reveals the presence of numerous spherical pits of various sizes. Figure 8; Figure 9 also contain the corresponding maps of the distribution of chemical elements on the scanned surface. Each map is represented in a different color which allows extracting the location of Fe and C elements originating from the Fe-C alloy and location of Sn, Sb, and Cl elements present on the surface due to etching. The maps show that all elements are homogeneously distributed in the investigated micro-regions; however, Fe in the areas corresponding to pits is present in a significantly lower amount due to corrosion process.

**Figure 10:**
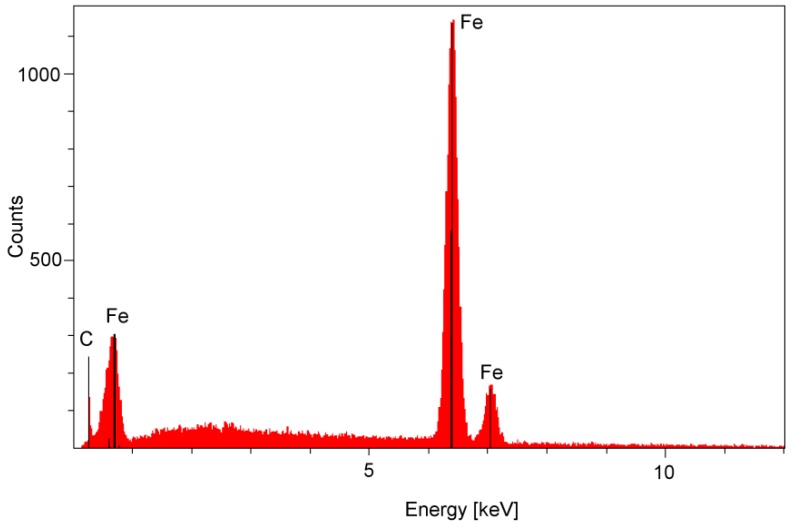
Energy dispersive spectrum in the micro-region of the AISI 1010 carbon steel surface after the experiment in sulfolane at 95 °C without etching and subjected to the potentiodynamic test.

A micro-analytical EDS technique conventionally used in SEM was also applied to locally determine chemical elements in the tested samples. The EDS spectra were collected from different locations on a homogeneous specimen with each single scan area of 10 × 10 μm^2^. A representative energy dispersive spectrum for the AISI 1010 carbon steel surface after the experiment in sulfolane at 95 °C without etching and subjected to the potentiodynamic test is presented in Figure 10. Presence of characteristic peaks from Fe and C components of the AISI 1010 carbon steel is observed in Figure 10.

**Figure 11:**
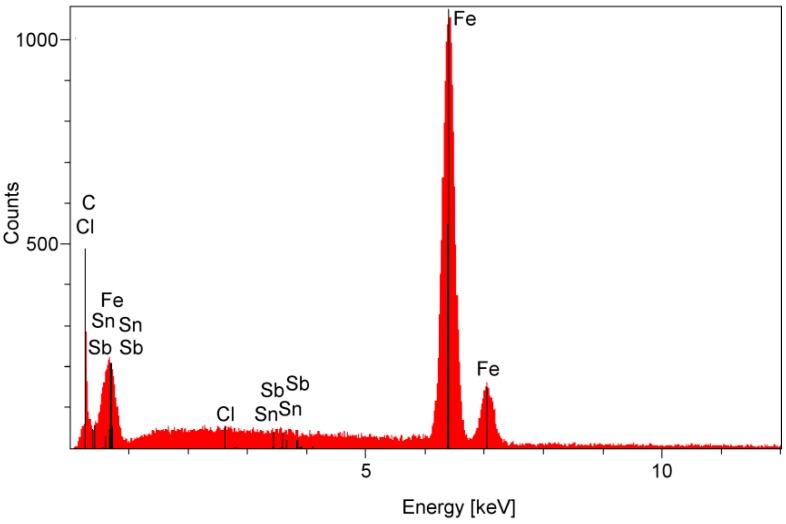
Energy dispersive spectrum in the micro-region of the AISI 1010 carbon steel surface after the experiment in sulfolane at 95 °C, etched, and subjected to the potentiodynamic test.

In the EDS spectrogram for the AISI 1010 carbon steel surface after the experiment in sulfolane at 95 °C, etched, and subjected to the potentiodynamic test (Figure 11), besides the spectral lines from alloying elements (Fe, C) some additional spectral lines from elements as Sn, Sb, and Cl are present which come from the etching solution.

**Figure 12:**
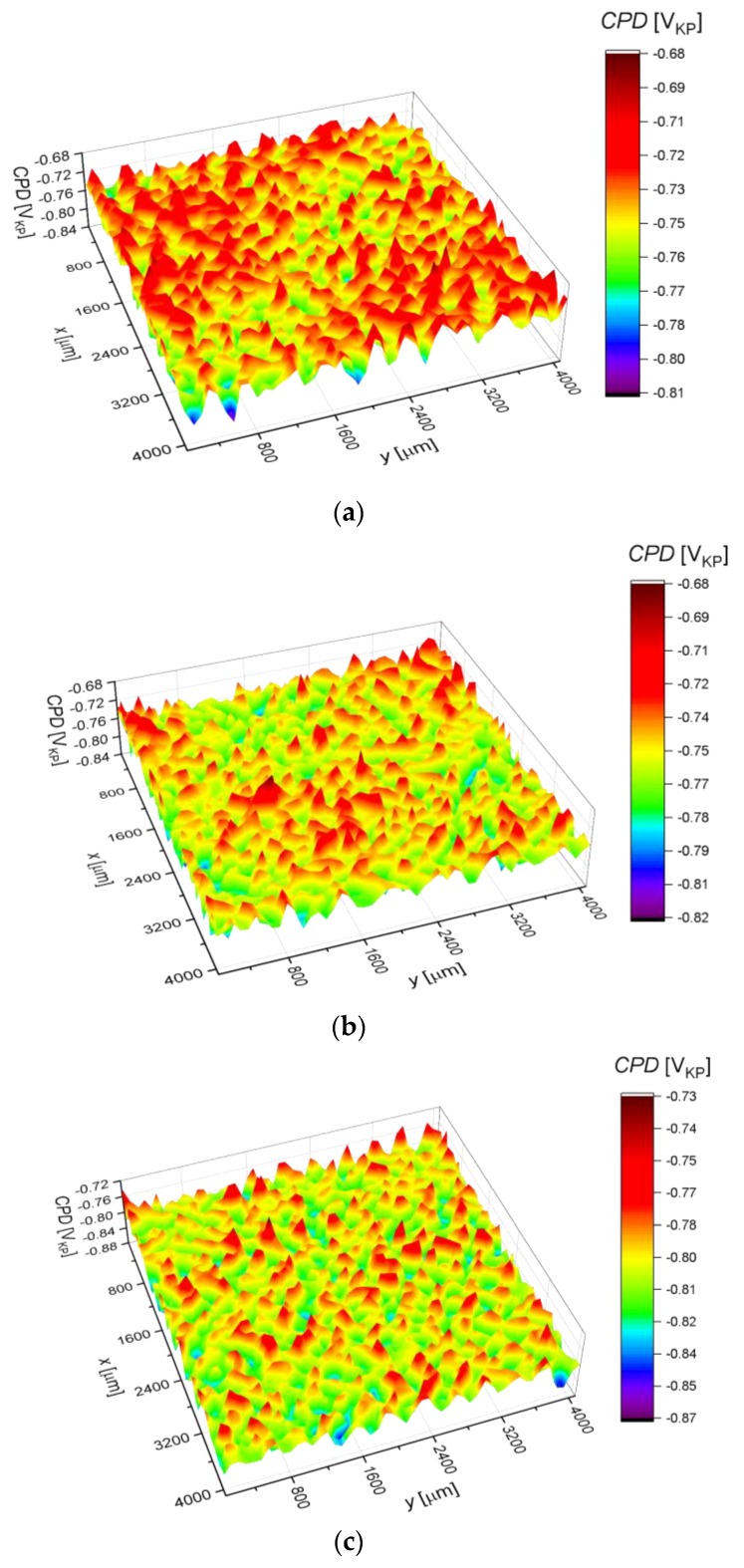

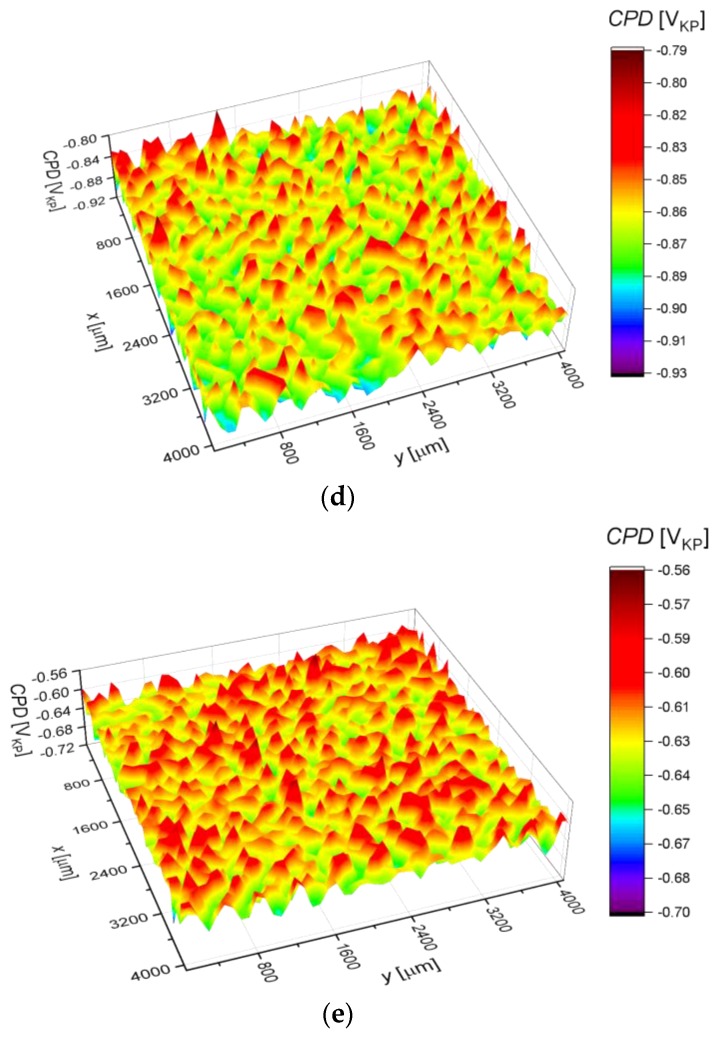
The *CPD* maps determined for the AISI 1010 carbon steel after the experiment in sulfolane at 95 °C and etching (**a**); the experiment in sulfolane with the addition of 1 vol.% of water at 95 °C and etching (**b**); the experiment in sulfolane with the addition of 2 vol.% of water at 95 °C and etching (**c**); the experiment in sulfolane with the addition of 3 vol.% of water at 95 °C and etching (**d**); after the experiment in sulfolane at 95 °C without etching (**e**).

**Figure 13:**
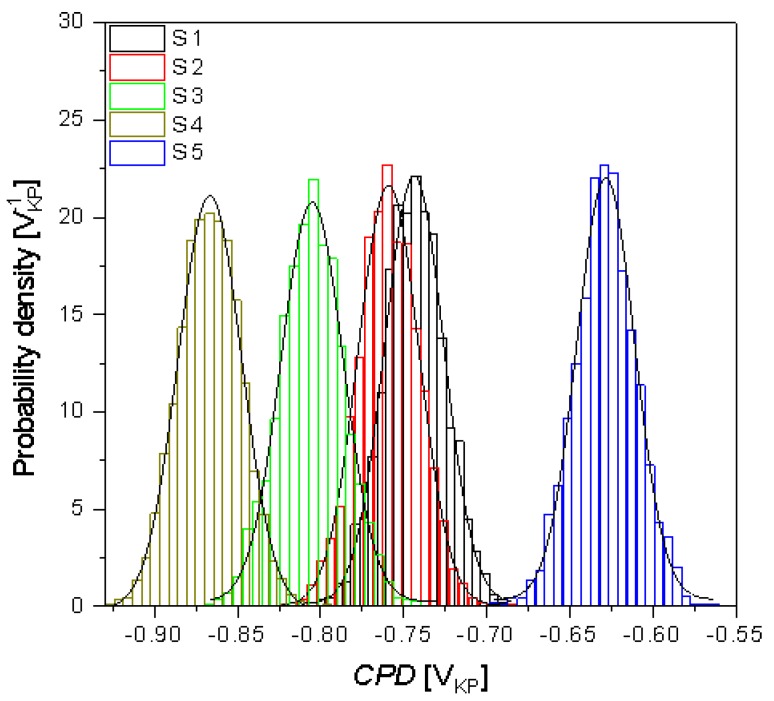
The *CPD* histograms determined for the AISI 1010 carbon steel after the experiment in sulfolane at 95 °C and etching (S1); the experiment in sulfolane with the addition of 1 vol.% of water at 95 °C and etching (S2); the experiment in sulfolane with the addition of 2 vol.% of water at 95 °C and etching (S3); the experiment in sulfolane with the addition of 3 vol.% of water at 95 °C and etching (S4); the experiment in sulfolane at 95 °C without etching (S5); solid lines—fit of the Gaussian distribution.

The contact potential difference maps (Figure 12) and corresponding histograms (Figure 13) were determined for the studied materials. Approximation of the obtained histograms by the continuous Gaussian function allows evaluating parameters that describe quantitatively the surface properties, i.e., average (*CPD*_av_) and root mean square (*CPD*_q_) summarized in Table 4.

It was found that the AISI 1010 carbon steel after the experiment in sulfolane at 95 °C and etching (Figure 12a) is characterized by the higher value of *CPD*_av_ of ca. −744 mV_KP_ in comparison with the tested samples: after the experiment in sulfolane with the addition of 1 vol.% of water at 95 °C and etching for which *CPD*_av_ is ca. −759 mV_KP_ (Figure 12b), after the experiment in sulfolane with the addition of 2 vol.% of water at 95 °C and etching for which *CPD*_av_ is ca. −805 mV_KP_ (Figure 12c), and after the experiment in sulfolane with the addition of 3 vol.% of water at 95 °C and etching with the *CPD*_av_ of ca. −867 mV_KP_ (Figure 12d). The AISI 1010 carbon steel after the experiment in sulfolane at 95 °C without etching (Figure 12e) is characterized by the highest *CPD*_av_ value (ca. −628 mV_KP_). This result may be related to the decreased porosity and/or roughness of the surface in comparison with the protective film present on the etched samples. It should also be noted that dispersion of *CPD* values around the mean (represented by *CPD*_q_) is the smallest for the S5 sample without etching and equals ca. 17.5 mV_KP_ (Table 4). This means that the corresponding sample shows the most homogeneous surface of all the samples tested.

**Figure 14:**
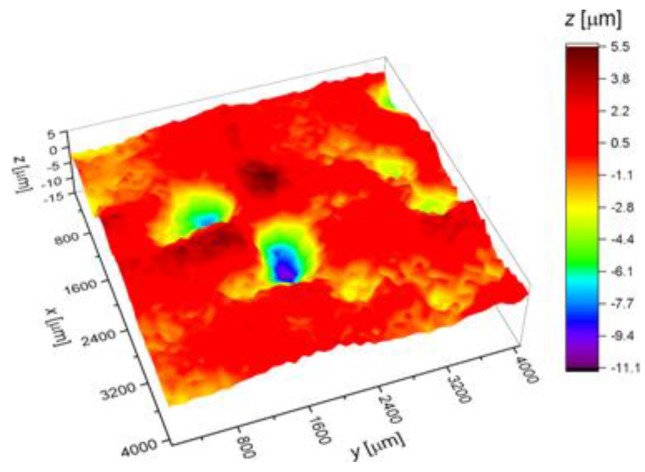
Topography map determined for the AISI 1010 carbon steel after the experiment in sulfolane at 95 °C and etching.

Figure 14 shows an exemplary surface topography map obtained for the AISI 1010 carbon steel following the experiment in sulfolane at 95 °C and etching. Statistical analysis of the obtained maps allows determining parameters that describe quantitatively the surface roughness, i.e., root mean square roughness (*S*_q_), maximum peak height (*S*_p_), and maximum pit depth (*S*_v_).

It was found that for the AISI 1010 carbon steel after the experiment in sulfolane at 95 °C and etching, pits of various diameters and depths were found which is in accordance with the SEM results (see Figure 7c). It was estimated that the diameter of pits changes from ca. 300 to 550 μm. Moreover, the statistical results obtained (i.e., *S*_q_ = 2.0 μm, *S*_p_ = 5.5 μm, and *S*_v_ = 11.1 μm) indicate large fluctuation in the heights of peaks and valleys and that the largest pits are about 11 μm deep.

## 4. Conclusions

1. The real-time corrosion monitoring with the metal surface mapping procedures were combined together in the pilot-case workflow for the exhaustive assessment of the sulfolane corrosive potential against AISI 1010 carbon steel. Several aspects of corrosion evaluation of the general and localized corrosion modes were examined using a dedicated testing vessel (our own design).
2. In fact, a noticeable influence of water (even at low temperature) on sulfolane corrosivity was observed; an increase in water concentration accelerates sulfolane degradation, as indicated by elevated corrosion rate and an increase in the suspended dark deposits. On the other hand, no detectable impact on carbon steel localized corrosion propensity was noted in the water-sulfolane mixtures.
3. The evaluation of the corrosion resistance of AISI 1010 carbon steel after the experiment in sulfolane with the addition of 1–3 vol.% of water at 95 °C in the absence and in the presence of the layer of corrosion products was conducted in pure sulfolane at 25 °C using the open circuit potential method and the potentiodynamic measurements. The estimation of the corrosion rate, polarization resistance, and Stern–Geary constant were made according to the ASTM G102 - 89(2015)e1. Based on the determined parameters of the corrosion resistance it was noticed that the increase in the water content (1–3 vol.%) in sulfolane affects the decrease in the corrosion resistance of AISI 1010 carbon steel on uniform and pitting corrosion due to higher conductance of the electrolyte.
4. It was found that the highest corrosion resistance among all tested electrodes revealed the electrode after previous experiment in sulfolane at 95 °C without etching of the layer of corrosion products with barrier properties. The evaluation of the corrosion damage for this electrode revealed the lowest value of the corrosion degree determined using the numerical analysis of sample surface images obtained with SEM, and the highest value of average contact potential difference specified using the scanning Kelvin probe technique.

## Figures and Tables

**Table 1:** Description of the recorded corrosion parameters.

Parameter	Definition
General corrosion rate (PV)	Measurement of the real part of the Low Frequency Impedance (LFI) of the working electrode. SmartCET uses Linear Polarization Resistance (LPR) technique to calculate the General Corrosion Rate that is usually the prime variable of interest, because it reflects the overall rate of metallic corrosion. Corrosion may be directly related to operational parameters, e.g., temperatures, flow, chemical composition.
Pitting Factor (PF)	Ratio of the depth of the deepest pit (point or small area, that takes the form of cavities) resulting from corrosion divided by the average penetration as calculated from weight loss. It is a measure of the overall stability of the corrosion process obtained from a measurement of the intrinsic current noise of the working electrode and comparing this measurement to the general corrosion obtained from the LPR measurement.
Dynamic B value	Corrosion constant also known as Stern–Geary constant. It is an essential part of the corrosion rate calculation that is directly proportional to the corrosion rate value. It represents a correction factor constant determined by the mechanism/kinetics of the corrosion process. In a dynamic process the B value is not constant. The knowledge of the B value enables to refine the LPR-generated corrosion rate estimation, since the uncertainty regarding the standard (default) B value is removed. The B value is directly related to the mechanistic properties of the component anodic and cathodic corrosion processes.
Corrosion mechanism indicator (CMI)	The CMI is a qualitative indicator of a surface film presence. If there is no film and only corrosion is present, the CMI will have an intermediate value. Inorganic scale, or thick passive oxide films with little or no conductivity, will show a low CMI value.

**Table 2:** Description of the analyzed AISI 1010 carbon steel samples in sulfolane-containing fluids.

Shape	Material	Temperature	Electrode	Surface Corrosion Sampling
3 × flat coupon electrode 89 mm × 20 mm × 2 mm	AISI 1010 carbon steel	95 °C	S1	The corrosion test and etching
			S2	The corrosion test with the addition of 1 vol.% of water and etching
			S3	The corrosion test with the addition of 2 vol.% of water and etching
			S4	The corrosion test with the addition of 3 vol.% of water and etching
			S5	The corrosion test without etching of corrosion products

**Table 3:** The parameters of corrosion resistance for the AISI 1010 electrodes in sulfolane at 25 °C determined based on analysis of the Tafel plots shown in Figure 5 and according to ASTM G102-89(2015)e1: Standard Practice for Calculation of Corrosion Rates and Related Information from Electrochemical Measurements.

Electrode No.	*E*_cor_ (V)	*j*_cor_ (A cm^−2^)	*b*_a_ (V dec^−1^)	*b*_c_ (V dec^−1^)	*B* (V)	*R*_p_ (cm^2^)	*CR* at *E*_cor_ (mpy)
S1	−0.016 ± 5.20 × 10^−4^	3.10 × 10^−8^ ± 4.40 × 10^−9^	0.562 ± 0.084	0.398 ± 0.048	0.101	3.26 × 10^6^	0.014
S2	−0.022 ± 5.54 ×10^−4^	1.16 × 10^−8^ ± 1.59 × 10^−9^	0.626 ± 0.111	0.298 ± 0.031	0.088	7.56 × 10^6^	0.005
S3	−0.0 07 ± 7.76 × 10^−4^	2.92 × 10^−8^ ± 1.21 × 10^−8^	0.478 ± 0.168	0.584 ± 0.268	0.114	3.91 × 10^6^	0.013
S4	−0.185 ± 4.98 × 10^−4^	1.46 × 10^−8^ ± 6.45 × 10^−10^	0.548 ± 0.021	0.387 ± 0.016	0.098	6.75 × 10^6^	0.007
S5	−0.104 ± 3.64 × 10^−4^	4.21 × 10^−9^ ± 2.30 × 10^−10^	0.777 ± 0.046	0.485 ± 0.022	0.130	3.08 × 10^7^	0.002

**Table 4:** Statistical parameters obtained for the contact potential difference (*CPD*) maps of the AISI 1010 carbon steel after the experiment in sulfolane at 95 °C and etching (S1); the experiment in sulfolane with the addition of 1 vol.% of water at 95 °C and etching (S2); the experiment in sulfolane with the addition of 2 vol.% of water at 95 °C and etching (S3); the experiment in sulfolane with the addition of 3 vol.% of water at 95 °C and etching (S4); and after the experiment in sulfolane at 95 °C without etching (S5); indexes: av and q refer to average and root mean square value; *V*_KP_ is the voltage measured in relation to the Kelvin probe.

Parameter	S1	S2	S3	S4	S5
*CPD*_av_ (mV_KP_)	−743.7(4)	−759.1(4)	−805.1(4)	−866.7(2)	−628.3(4)
*CPD*_q_ (mV_KP_)	17.8(1)	18.3(9)	18.7(9)	19.2(7)	17.5(1)
